# *Polygonum hydropiper* extract attenuates ethanol-induced gastric damage through antioxidant and anti-inflammatory pathways

**DOI:** 10.1590/1414-431X2020e10841

**Published:** 2021-05-24

**Authors:** Shouzhong Ren, Bangpei Chen, Zhijian Ma, Hui Hu, Yiqiang Xie

**Affiliations:** 1 Key Laboratory of Tropical Translational Medicine of Ministry of Education, Hainan Provincial Key Laboratory for Research and Development of Tropical Herbs, School of Pharmacy, Hainan Medical University, Haikou, Hainan, China; 2 Key Laboratory of Ministry of Education for Advanced Materials in Tropical Island Resources, Hainan University, Haikou, Hainan, China; 3 College of Traditional Chinese Medicine, Hainan Medical University, Haikou, Hainan, China

**Keywords:** Polygonum hydropiper, Gastric mucosal lesions, Flavonoids, Antioxidative, Anti-inflammatory

## Abstract

The present study was conducted to investigate the underlying mechanisms and effective components of *Polygonum hydropiper* in ethanol-induced acute gastric mucosal lesions. The ethanol extract was purified on an AB-8 macroporous resin column and eluted with 60% ethanol and was then injected into the HPLC system for quantitative analysis. Sprague-Dawley rats were orally pretreated with *P. hydropiper* extract (PHLE; 50, 100, and 200 mg/kg) for 5 days and then absolute ethanol was administered to induce gastric mucosal damage. One hour after ethanol ingestion, the rats were euthanized and stomach samples were collected for biochemical analysis. Antioxidant enzymes and anti-inflammatory cytokines were quantified. Western blotting was used to detect the expression levels of proteins. Cell proliferation was assayed by CCK-8 assays. The proportion of total flavonoids in the final extract of *P. hydropiper* was 50.05%, which contained three major bioactive flavonoid constituents, including rutin, quercitrin, and quercetin. PHLE significantly increased cell viability and effectively protected human gastric epithelial cells-1 against alcohol-induced damage *in vitro*. PHLE pretreatment attenuated gastric mucosal injuries in a dose-dependent manner in rats, and increased the activity of superoxide dismutase, glutathione peroxidase, and glutathione, and decreased the levels of malondialdehyde in gastric tissue. Pretreatment with PHLE also reduced the generation of the pro-inflammatory cytokines tumor necrosis factor-α and interleukin-1β in gastric tissue by downregulating the expression of nuclear factor-kappa B. PHLE exerted protective effects against gastric injury through antioxidant and anti-inflammatory pathways. Flavonoids might be the main effective components of *P. hydropiper* against gastric mucosal injury.

## Introduction

Acute gastric damage is a common gastrointestinal disease that affects many people worldwide and is frequently caused by excessive alcohol consumption, prolonged nonsteroidal anti-inflammatory drug use, or stress (1,2). The morbidity rate associated with the gastric injuries induced by extreme intake of alcohol is increasing each year. Thus, it is an important public health problem to relieve gastric illness, including stomach ulcers, caused by alcohol.

Ethanol, as an exogenous irritant, is known to lead to acute gastric mucosal lesions. Excessive intake of some alcoholic drinks can also result in human gastric damage, and the degree of injury is closely related to ethanol concentration and quantity (3,4). Ethanol not only directly injures gastric mucosal cells but also sensitizes the gastric mucosa to injury (5). Studies have shown that an imbalance of aggressive and defensive factors is the basic mechanism of the development of gastric damage. Inflammatory mediators and reactive oxygen species (ROS) are two important offensive factors in the pathogenesis of acute gastric mucosal lesions induced by ethanol (6–8). Pro-inflammatory cytokines, including interleukin (IL)-1β, IL-6, and tumor necrosis factor (TNF)-α, cause inflammatory reactions and contribute to ROS generation during the inflammatory process, while ROS not only directly damage cell structures but also promote the production of pro-inflammatory factors. During the development of gastric injury, pro-inflammatory cytokines and ROS influence and mutually promote injury, leading to aggravation of the damage. Therefore, anti-oxidation and anti-inflammation play an important role in protecting the gastric mucosa against damage.

The traditional Chinese herb *Polygonum hydropiper* Linn (Polygonaceae) has been considered for treating gastric injury due to its long history in the treatment of gastrointestinal diseases without obvious adverse effects (9). It has the function of dispelling dampness, removing toxins, dissipating blood stasis, and relieving pain in traditional Chinese medicine. Flavonoids are the main component in *P. hydropiper* ethanol extract (10), which has anti-inflammatory, antioxidant, and analgesic effects (11). This extract can inhibit neutrophil infiltration, decrease pro-inflammatory factor levels (such as TNF-α and IL-1β), and increase the activity of antioxidant enzymes, as indicated by a molecular pharmacology study (12). We previously showed that *P. hydropiper* water extract has a preventive effect on gastric mucosa injury (13), and further study showed that the effect of ethanol extract was obviously superior to that of water. However, the mechanisms of *P. hydropiper* ethanol extract in the treatment of gastric illness remain unknown. Thus, the purpose of this study was to evaluate the effect of an ethanol extract from *P. hydropiper* on ethanol-induced gastric mucosa injury in rats and to explore the underlying mechanisms.

## Material and Methods

### Chemicals and reagents

Superoxide dismutase (SOD), malondialdehyde (MDA), glutathione peroxidase (GSH-Px), and glutathione (GSH) were purchased from Jiancheng Biotech (China). Anti-nuclear factor-kappa B (NF-κB) p65 was purchased from Abcam (USA). Enzyme-linked immunosorbent assay (ELISA) kits for TNF-α and IL-1β were purchased from ExCell Biotech (China).

### Experimental animals

Sprague-Dawley rats (6 weeks old) were purchased from Changsha Tianqing Biotech Co., Ltd. (China). All animals were maintained in a room at 25°C under a 12-h light/dark cycle, with free access to food and water. After 1 week of acclimatization, the rats were used for experiments. All procedures involving animals were performed in accordance with the international Guidelines for Care and Use of Laboratory Animals and were approved by the Animal Ethics Committee of Hainan Medical University (201506017/HMU).

### Plant material and preparation of PHLE

The stem and leaves of *P. hydropiper* were collected from Hainan Province in China, and plant identity was confirmed by Prof. Niankai Zeng of Hainan Medical University. Voucher specimens (No. 20191016) were deposited at the School of Pharmacy, Hainan Medical University. The stems and leaves of the plants were air-dried at room temperature. The material was extracted with 10-fold ethanol solvent (60%) for 1 h after grinding into powder. The residue was extracted twice under the same conditions. The samples were filtered, combined, and concentrated under reduced pressure. Then, the extract was purified on an AB-8 macroporous resin column and initially eluted with 2000 mL of water and then with 60% ethanol. The water elution was discarded and the ethanol elution remained.

### High performance liquid chromatography

Chromatographic experiments were performed on a Waters e2695 LC System. X Bridge C18 column (250×4.6 mm, 5 µm; Waters Co., Ltd., USA) was used as the stationary phase. The mobile phase was composed of acetonitrile-0.2% acetic acid (35:65, v/v). The system was equilibrated for 20 min with the starting conditions, then the mobile phase was used to elute for 40 min. The flow rate was maintained at 1.0 mL/min. The column temperature was maintained at 30°C and the injection volume was 20 µL. The detection wavelength was 355 nm. The standard stock solutions of rutin, hyperoside, quercetin, and quercitrin were dissolved with methanol and filtered through a 0.45-µm membrane prior to injection.

### Cell culture and treatment

Human gastric epithelial cells (GES-1) were purchased from the Beijing Institute of Cancer (China). GES-1 cells were cultured in 1640 medium containing 10% fetal bovine serum and 1% penicillin/streptomycin in an incubator with 5% CO_2_ at 37°C until 80% confluency was reached. The cells were then trypsinized and subcultured. When cells reached 70-80% confluence, they were pretreated with various concentrations of PHLE (10, 20, and 40 μg/mL) in complete 1640 medium for 12 h, followed by stimulation with 7% ethanol for 4h.

### Cell viability assay

The GES-1 cells were incubated on a 96-well plate (1×10^4^ cells/well) and cultured for 24 h after the addition of 1640 medium. CCK-8 reagent (Dojindo, Japan) was added to the different groups at various concentrations. The reagent was mixed with 1640 medium at a ratio of 1:9, and then 100 μL of the solution was quickly added to each well after mixing. The plate was incubated at 37°C for 4 h, and the absorbance value of each well was detected at 450 nm with an assay reader (Multiskan GO, Thermo Fisher, USA).

### Animal model and treatment

Rats were randomly divided into six groups (n=10 per group, both sexes): the normal control group, model group, positive group, and 3 *P. hydropiper* extract (PHLE 50, 100, and 200 mg/kg) groups. The rats were orally administered PHLE for 5 days at doses of 50, 100, and 200 mg/kg body weight. The rats in the normal control and model groups were administered an equivalent volume of distilled water, while those in the positive control group were orally administered ranitidine (50 mg/kg, Shiyao Group, China). The rats were fasted for 24 h after the fifth day of gavage. On the 6th day, all groups of rats, except the normal control group, were challenged orally with anhydrous ethanol (99.5%) at a dose of 10 mL/kg, while those in the normal control group were given an equivalent volume of distilled water. One hour after ethanol administration, the rats were euthanized under deep anesthesia with pentobarbital sodium (50 mg/kg, *ip*), and the stomach was immediately removed and opened through the large curvature to macroscopically observe the level of injury.

### Sample collection

Gastric samples were washed using ice-cold saline. Then, a part of the stomach was used to prepare tissue homogenate (10%) on ice with PBS (phosphate-buffered saline) buffer, and the homogenate was centrifuged at 14,000 *g* for 15 min at 4°C. The supernatants were collected and stored at −80°C until biochemical analysis. Another portion of gastric samples was also weighed, frozen, and kept at -80°C for subsequent western blot analysis.

### Gross ulcer index

The mucosa of the stomachs was photographed using a digital camera and gastric damage, namely, macroscopic lesions, was observed under a dissecting microscope. The severity of gastric mucosal injury was estimated using a gross ulcer index (GUI). The length (mm) of each injury was measured and the injury index was calculated as the sum of the length of all injuries. The length per 1 mm was recorded as a score of 1. If the width was more than 1 mm, the score was doubled and normal stomachs received a score of 0.

### Analysis of SOD, GSH-Px, and MDA

The tissue concentration of MDA was measured using thiobarbituric acid. The activity of SOD, GSH, and GSH-Px in tissue was analyzed using commercially available kits according to the manufacturer's instructions (Nanjing Jiancheng Biotech, China).

### Cytokine assays

The levels of TNF-α and IL-1β in gastric mucosa were measured by ELISA kits following the manufacturer's instructions. Protein concentrations were measured using the BCA assay kit (Beyotime, China) and a BioTek microplate reader (BioTek Instruments, Inc., USA).

### Western blot analysis

Mucosa specimens were rapidly scraped from underlying gastric tissue layers using two glass slides, which were kept on ice. The mucosal tissues were weighed, minced by ophthalmic scissors, and homogenized in radioimmunoprecipitation assay (RIPA) lysis buffer containing protease and phosphatase inhibitor mixture (1% phenylmethanesulfonyl fluoride (PMSF) and cocktail; Beyotime). The total protein concentration was measured using a BCA protein assay kit (Beyotime) and a BioTek microplate reader. Protein extracts (30 µg) were separated with 10% sodium dodecyl sulfate-polyacrylamide gel electrophoresis (SDS-PAGE), and then the proteins were transferred onto polyvinylidene difluoride (PVDF) membranes (Millipore, USA). The blots were blocked with 5% skim milk in Tris-buffered saline Tween-20 (TBST) for 1 h and subsequently probed overnight at 4°C with rabbit polyclonal anti-NF-κB p65 (1:2000) primary antibodies (Abcam, UK). Then, the blots were washed with Tris-buffered saline containing Tween-20 and incubated with a goat anti-rabbit horseradish peroxidase (HRP)-conjugated secondary antibody (Abcam) for 1.5 h at room temperature. The bands were observed using an enhanced chemiluminescence substrate, and protein expression was quantified using a fully automated image analysis system (Tanon 4500 s; Tanon Technology Co., Ltd., China). 

### Statistical analysis

The data are reported as means±SD. The statistical analyses were performed by one-way analysis of variance (ANOVA) and the least significance difference (LSD) *post hoc* test using SPSS 20.0 software (IBM, USA). P<0.05 was considered statistically significant in all cases.

## Results

### HPLC analysis of PHLE

**Figure 1:**
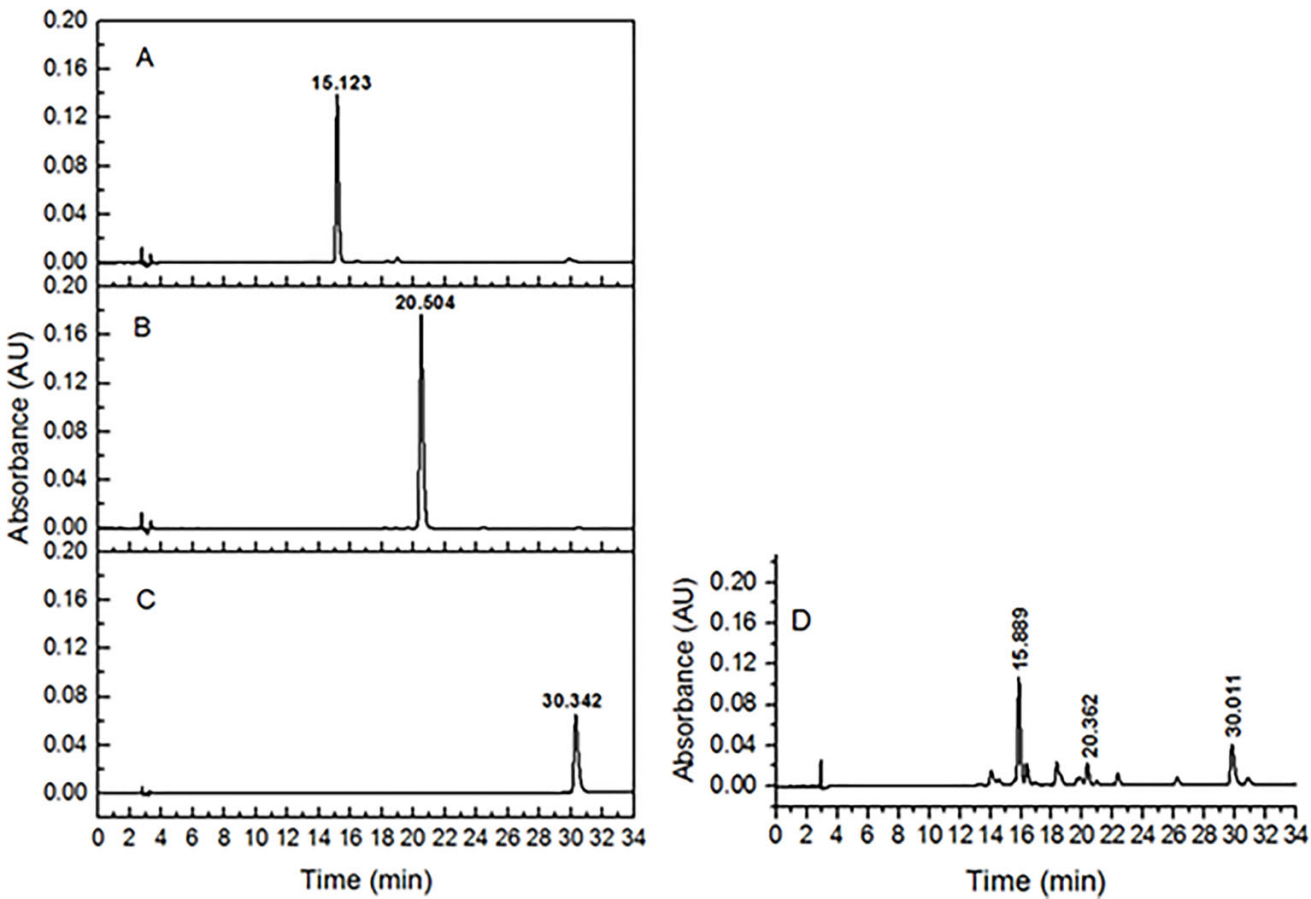
HPLC chromatograms of rutin (**A**), quercitrin (**B**), quercetin (**C**), and PHLE extract (**D**). PHLE: *P. hydropiper* extract; HPLC: high-performance liquid chromatography.

The results of the high-performance liquid chromatography (HPLC) analysis of PHLE are shown in the chromatograms in Figure 1. The peaks of marker substance in PHLE (Figure 1D) were identified according to the retention times of their reference samples (Figure 1A-C). The proportion of flavonoids in the final extract was more than 50%. The extract contained three major bioactive flavonoid constituents, including rutin (25.43%), quercitrin (7.08%), and quercetin (17.54%).

### PHLE promoted cell proliferation

**Figure 2:**
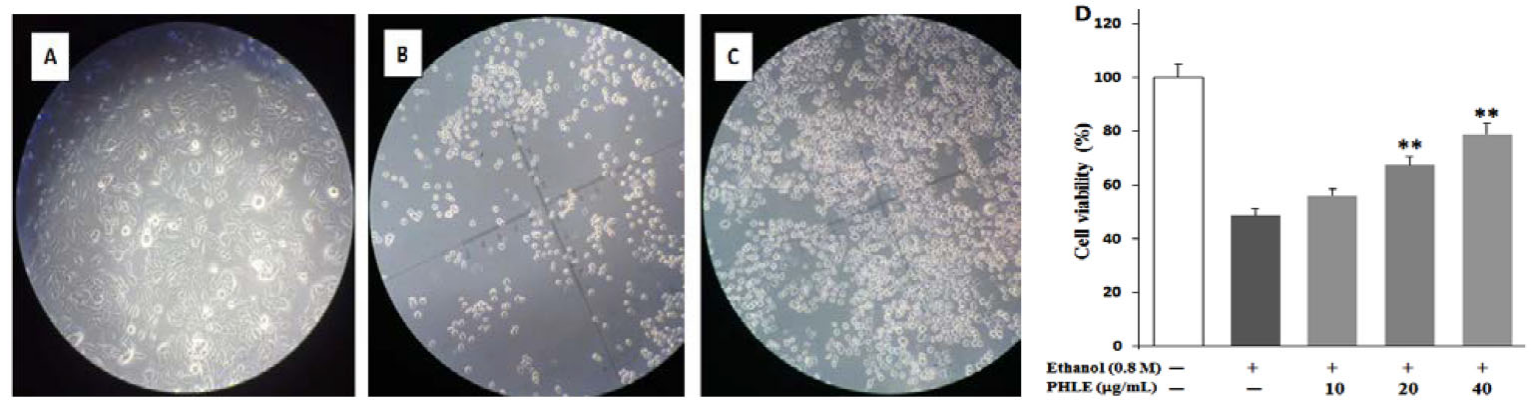
*P. hydropiper* extract (PHLE) enhanced the viability of GES-1 cells. **A**, normal group; **B**, ethanol group; **C**, PHLE (40 μg/mL) group. Cells were pretreated with various concentrations of PHLE for 12 h followed by stimulation with 7% ethanol for 4 h. **D**, Cell viability was determined by CCK-8 assay. Data are reported as means±SD (n=6). **P<0.01 compared to the ethanol group (ANOVA and LSD *post hoc* test).

The results confirmed that cell viability changed significantly after treatment with different concentrations PHLE. The activity of the cells decreased to 48.53% after stimulation with 0.8 M ethanol. After 12 h of PHLE treatment, the cell viability increased from 49.02 to 78.86%, and the effect was dose-dependent (Figure 2).

### General observations and ulcer index of animal model

**Figure 3:**
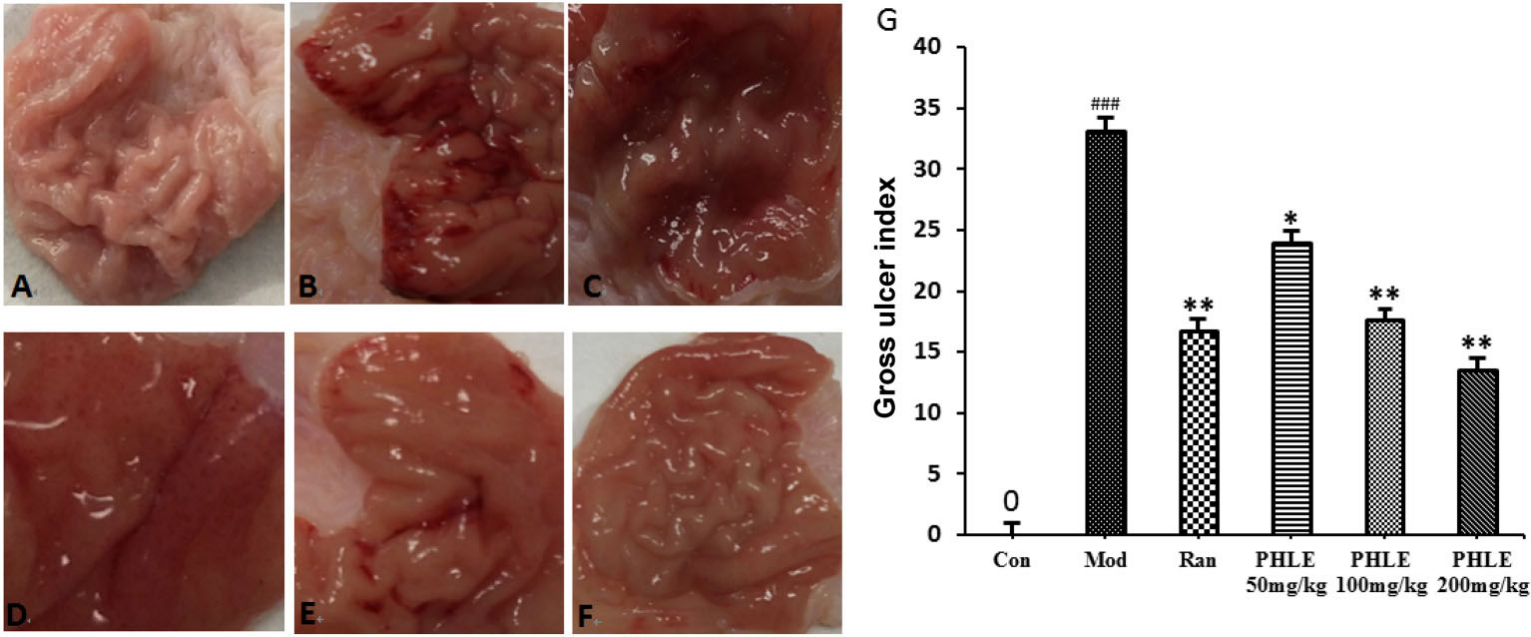
Gross findings of ethanol-induced gastric damage in the rat gastric mucosa. The rats were pretreated with different doses of *P. hydropiper* extract (PHLE) or ranitidine (Ran). After 1 h, anhydrous ethanol (10 mL/kg) was administered orally. One hour after EtOH administration, the rats were euthanized to evaluate the gross morphology of the gastric mucosa. **A**: normal control group; **B**: model group; **C**: ranitidine group; **D**: PHLE 50 mg/kg; **E**: PHLE 100 mg/kg; **F**: PHLE 200 mg/kg; **G**: Gross ulcer index. Data are reported as means±SD. ^###^P<0.001 compared to the normal control (Con) group; *P<0.05 and **P<0.01 compared to the model (Mod) group (ANOVA and LSD *post hoc* test).

After the administration of anhydrous ethanol, visible mucosal shedding, necrosis, edema, and hyperemia or hemorrhage were observed in the model group compared to the normal control group. The *P. hydropiper* extract groups showed significantly decreased gastric mucosal injury to varying degrees compared to the model group (P<0.05 or P<0.01, Figure 3), which indicated that edema, erosion, and hemorrhage or ecchymosis were significantly ameliorated in the gastric mucosa and that the extract demonstrated a concentration-dependent protective effect. The GUI was increased significantly in the model group compared with the normal control group. A significant reduction in the GUI was observed in the PHLE 200 mg/kg and 100 mg/kg groups compared with the model group (P<0.01) (Figure 3).

### Effects of PHLE on antioxidant activity

**Figure 4:**
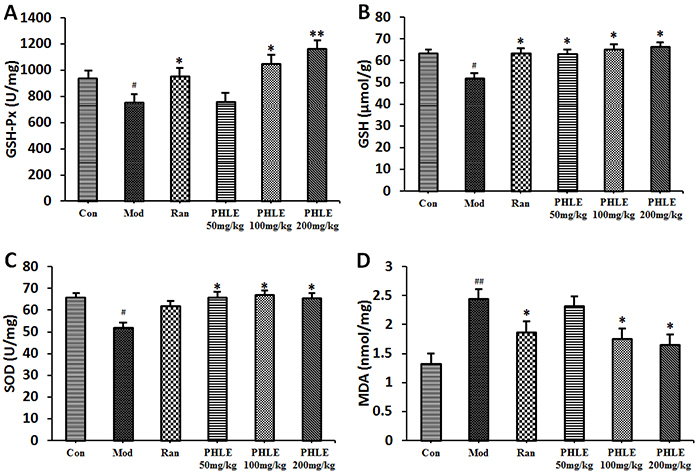
Effects of *P. hydropiper* extract (PHLE) on GSA-Px (**A**), GSH (**B**), and SOD (**C**) activity and MDA level (**D**) in gastric tissues from rats with ethanol-induced gastric damage. PHLE (50, 100, and 200 mg/kg) was used to pretreat rats. The data are reported as means±SD (n=10). ^#^P<0.05 and ^##^P<0.05 compared to the normal control group (Con); *P<0.05 and **P<0.01 compared to the model group (Mod) (ANOVA and LSD *post hoc* test). Ran: ranitidine (positive control).

After ethanol challenge, the antioxidant enzyme activity decreased in the model group compared with the normal control group, as evidenced by decreased levels of SOD, GSH, and GSH-Px. In the PHLE groups, after 5 consecutive days of treatment with PHLE, the activity of SOD, GSH, and GSH-Px was significantly elevated. Although the antioxidant capacity of PHLE was not dose-dependent, it was not significantly different between the PHLE 200 mg/kg and PHLE 100 mg/kg groups. The PHLE groups and the normal control group had similar levels of SOD and GSH, and enzyme activity was not significantly different among the PHLE groups. However, the activity of GSH-Px in the three groups was significantly different, and the activity in the 50 mg/kg group was significantly lower than that in the other two groups. In addition, the PHLE 50 mg/kg group and the model group had similar levels of MDA, but MDA levels decreased significantly in the PHLE 200 mg/kg and PHLE 100 mg/kg groups compared with the PHLE 50 mg/kg and model groups (Figure 4).

### Effects of PHLE on the levels of inflammatory cytokines

**Figure 5:**
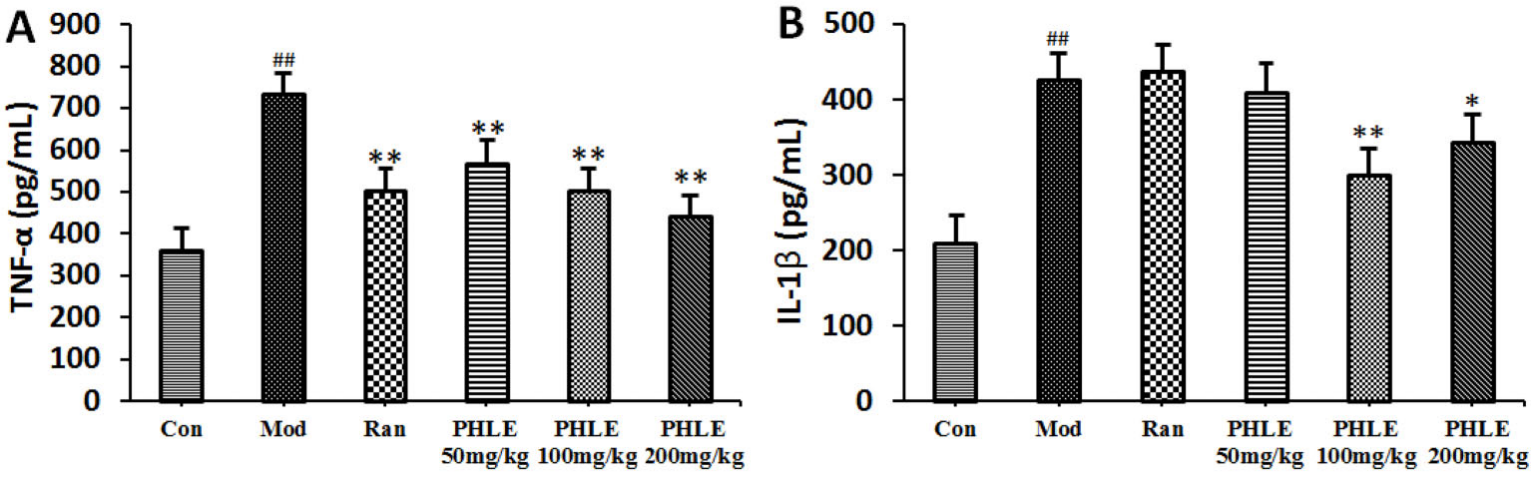
Effects of *P. hydropiper* extract (PHLE) on tumor necrosis factor (TNF)-α (**A**) and interleukin (IL)-1β (**B**) levels in the gastric tissues of rats with ethanol-induced gastric damage. The data are reported as means±SD (n=10). ^##^P<0.01 compared to the normal control group (Con), *P<0.05 and **P<0.01 compared to the model group (Mod) (ANOVA and LSD *post hoc* test). Ran: ranitidine (positive control).

As shown in Figure 5, the levels of TNF-α and IL-1β were increased significantly in the gastric mucosal tissue of the model group compared with that of the normal control group. PHLE-treated rats showed a marked decrease in the excessive release of TNF-α compared with the model rats, and there were no differences among the PHLE groups. However, the results showed that the inhibitory effect of PHLE on IL-1β was very different among the PHLE groups, and the content of IL-1β in the PHLE 200 mg/kg and PHLE 100 mg/kg groups were significantly decreased while those in the PHLE 50 mg/kg group were not significantly different from those in the model group (Figure 5).

### Effects of PHLE on NF-&mac_kgr;B protein expression

**Figure 6:**
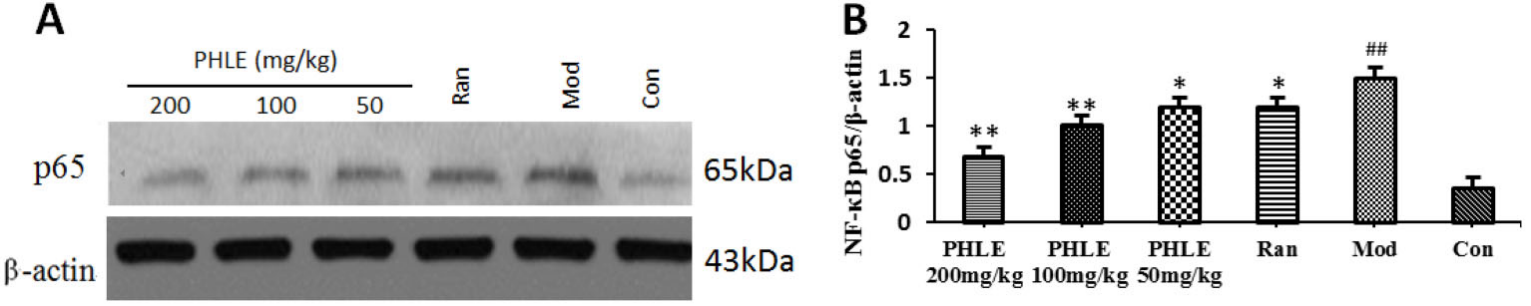
Effects of *P. hydropiper* extract (PHLE) on the total nuclear factor-kappa B (NF-κB) p65 level in the gastric tissues of rats with ethanol-induced gastric damage. **A**, Western blot analysis for protein expression of NF-κB p65; **B**, NF-κB p65/β-actin ratio. The data are reported as means±SD (n=10). ^##^P<0.01 compared to the normal control group (Con); *P<0.05 and **P<0.01 compared to the model group (Mod) (ANOVA and LSD *post hoc* test). Ran: ranitidine (positive control).

The gastric mucosal expression of NF-κB p65 protein was significantly increased in ethanol-induced rats. After 5 days of pretreatment with PHLE, the expression of NF-κB p65 protein among the groups pretreated with PHLE was significantly lower than that in the model group, indicating that PHLE downregulated p65 expression. The levels of NF-κB p65 were not significantly different in the PHLE 200 mg/kg and PHLE 100 mg/kg groups, but the NF-κB p65 levels in the PHLE 50 mg/kg group were lower than those in the other two groups (Figure 6).

## Discussion

As a traditional herbal medicine, *P. hydropiper* L has several biological activities. In the present study, the protective effect and mechanism of *P. hydropiper* L ethanol extract against acute gastric mucosal injury were assessed in ethanol-induced rat models. The results demonstrated that the administration of anhydrous ethanol caused macroscopic gastric mucosal injury and a significant increase in the GUI in the model rats. In contrast, PHLE significantly mitigated ethanol-induced gastric mucosal lesions and reduced the GUI. Similar results have also been reported with the crude methanol extract of *P. hydropiper* L having significant anti-ulcerogenic tendency and gastroprotective effect in aspirin-induced pyloric ligation ulcer model (14). Our data also showed that PHLE significantly promoted cell proliferation and increased cell viability *in vitro*, indicating it could effectively protect GES-1 cells against alcohol-induced damage.

Excessive ethanol intake is one of the main causes of acute gastric mucosal damage in humans (15,16); therefore, absolute ethanol administration is commonly used as an animal model of gastric injury in rats or mice. Oxidative stress and subsequent inflammation are the crucial pathogenic processes of the gastric injury provoked by ethanol administration (17). Ethanol can promote the production of ROS and the depletion of cellular antioxidant enzymes and can induce oxidative stress in many ways (18). Oxidative stress can contribute to the production of various pro-inflammatory mediators (19,20) and lead to inflammatory reactions (21). Subsequently, inflammatory reactions can also produce ROS, which lead to oxidative stress, and the interactions between them lead to the gradual formation of damage.

Previous studies have demonstrated that ethanol induces gastric mucosal injury by increasing the production of reactive oxygen radicals and subsequently oxidative stress (22). Therefore, scavenging free radicals and anti-oxidation can reduce the gastric mucosal damage induced by ethanol. SOD, GSH, and GSH-Px are the three main antioxidant enzymes involved in the elimination of oxygen radicals and act as an important antioxidant defense in gastric cells against ethanol-induced oxidative stress (23). Their activity can reflect the antioxidant capacity of gastric tissues and the influence of *P. hydropiper* L ethanol extracts on it, which may indirectly reflect the antioxidant capacity of *P. hydropiper*. MDA levels can be used to estimate the degree of lipid peroxidation of gastric tissues (24) and the effects of *P. hydropiper* on anti-lipid peroxidation in ethanol-induced gastric damage. Our results showed that ethanol administration significantly increased gastric injury and decreased the activity of antioxidant enzymes in gastric tissue, indicating that ethanol-induced gastric mucosal damage is negatively related to the decrease in SOD, GSH, and GSH-Px activity in gastric tissue, and this decrease in activity leads to the accumulation of ROS in gastric mucosal cells, increases lipid peroxidation, and eventually results in cell death. It has been reported previously that protective effects on the gastric mucosa was associated with increased SOD, GSH, and GSH-Px activity in treated groups compared to model groups (25).

After pretreatment with *P. hydropiper* ethanol extract, the activities of SOD, GSH, and GSH-Px were increased, and gastric injury was correspondingly reduced. The superoxide anions induced by ethanol are dismutated into H_2_O_2_ by SOD in gastric mucosal cells, which is then reduced to water by GSH-Px, suggesting that *P. hydropiper* ethanol extract could act as an antioxidant agent and alleviate the gastric mucosa damage induced by ethanol through free radical scavenging. *P. hydropiper* scavenges free radicals directly, mainly through direct chemical combination with free radicals, and indirectly, mainly through improving the activity of various antioxidant enzymes. Our data indicated that after the gastric mucosa was exposed to absolute ethanol for 1 h, MDA levels increased significantly in the gastric tissue of rats. The MDA level of the groups pretreated with *P. hydropiper* extract was decreased compared with that of the model group, suggesting that *P. hydropiper* extract can enhance the antioxidant capacity of the gastric mucosa, inhibit lipid peroxidation, and mitigate ethanol-induced damage to the stomach.

Inflammation is another crucial factor involved in the pathogenesis of ethanol-induced gastric mucosal lesions. Ethanol-induced gastric mucosal damage was accompanied by a significant increase in the content of pro-inflammatory cytokines, such as IL-1β, IL-6, and TNF-α. It has been shown that gastric epithelial cell apoptosis induced by ethanol is associated with an increase in mucosal TNF-α levels (26). Pro-inflammatory cytokines can induce the infiltration of neutrophils, trigger the production of additional inflammatory cytokines, and result in an inflammatory response that aggravates gastric tissue damage (27,28). Moreover, inflammatory cytokines may trigger oxidative pathways and produce ROS, which lead to oxidative stress in gastric tissue (29). Therefore, blocking the formation of inflammatory cytokines contributes to decreased inflammation and ROS production in the gastric mucosa, which alleviates gastric tissue damage. Nuclear factor-kappa B (NF-κB) is an important transcription factor that regulates the inflammatory response. NF-κB mediates the expression of pro-inflammatory cytokines and several other adhesion molecules (30,31). After ethanol administration, the expression of the pro-inflammatory cytokines TNF-α and IL-1β increased significantly in gastric tissue, indicating that TNF-α and IL-1β are involved in ethanol-induced gastric injury (32). Pretreatment with *P. hydropiper* extract significantly decreased the levels of TNF-α and IL-1β compared with those in the model group, suggesting that *P. hydropiper* extract had a good inhibitory effect on inflammation in gastric tissue. Similar results were also observed after butyrate treatment in ethanol-induced gastric mucosal lesions (33). We further evaluated the effect of *P. hydropiper* extract on the expression of NF-κB in gastric tissue, and the data showed that the administration of *P. hydropiper* extract greatly inhibited the expression of NF-κB. Some investigations have also shown that the aqueous extract of *P. hydropiper* has a protective effect on TNBS-induced intestinal inflammation in rats and its anti-inflammatory effects are closely related to inhibition of NF-κB signal pathways (34).

Flavones are the main component of *P. hydropiper* extract, and it has been reported that a variety of flavonoids possess obvious antioxidant and anti-inflammatory activity (35–37). Flavonoids isolated from the methanol extract of *P. hydropiper* leaves show strong antioxidant activity (38,39). The methanol extracts of *P. hydropiper* exert strong anti-inflammatory activity by suppressing the production of NO, TNF-α, NF-κB, and PGE_2_, and quercetin was found as one of the main active ingredients (40). Therefore, we hypothesized that flavones (rutin, quercetin, and quercitrin) might be responsible for the protective effect on gastric mucosal injury. Although this plant has long been used as a traditional herbal medicine for treatment of gastrointestinal diseases in the south of China, the effective components of its protective effects against gastric injury has rarely been reported. We intend to further investigate the chemical components of PHLE and combine it with pharmacodynamic experiments to identify the protective effect of this plant on gastric mucosa.

In conclusion, the results of the current study demonstrated that *P. hydropiper* extract alleviated ethanol-induced gastric mucosal injury in rats by mainly increasing antioxidant enzyme activity to eliminate ROS, decrease lipid peroxidation, and inhibit the formation of pro-inflammatory cytokines to relieve inflammatory reactions. Our findings suggest that *P. hydropiper* suppressed the production of inflammatory mediators by regulating NF-κB signaling pathways. Flavonoids might be the main effective components of *P. hydropiper* against gastric mucosal injury. Further studies are needed to elucidate the antioxidative mechanism in gastric protection and the active ingredients of *P. hydropiper*.
